# Effects of Fermented Rapeseed Meal as a Substitute for Soybean Meal on Growth Performance, Nutrient Digestibility, Serum Biochemical Indices and Gastrointestinal Microbiota of Sika Deer (*Cervus nippon*) During the Pre-Antler Growth Period

**DOI:** 10.3390/ani16081221

**Published:** 2026-04-16

**Authors:** Jiaxin Tian, Hui Zhao, Qiaoru Zhang, Haoran Sun, Zuer Gao, Luyang Sun, Chengzhi Zhu, Fansheng Kong, Xiuhua Gao, Qingkui Jiang, Tietao Zhang

**Affiliations:** 1 Institute of Special Animal and Plant Sciences, Chinese Academy of Agriculture Sciences, Changchun 130112, China; tjx0813@126.com (J.T.); baobeihuihui815@163.com (H.Z.); sarahzhang96@163.com (Q.Z.); solomoncat@163.com (H.S.); gze1010@163.com (Z.G.); 17767877397@163.com (L.S.); 13174428551@163.com (C.Z.); 15562382821@163.com (F.K.); 2 Institute of Feed Research, Chinese Academy of Agriculture Sciences, Beijing 100081, China; xiuhuagao@126.com; 3 Public Health Research Institute, New Jersey Medical School, Rutgers Biomedical and Health Sciences, Rutgers, The State University of New Jersey, Newark, NJ 07103, USA; 4 National Nanfan Research Institute, Chinese Academy of Agricultural Sciences, Sanya 572100, China

**Keywords:** soybean meal, fermented rapeseed meal, substitution, sika deer, pre-antler growth period

## Abstract

This study investigated the effects of replacing soybean meal with fermented rapeseed meal (FRSM) in the diets of sika deer (*Cervus nippon*) during the pre-antler growth period. A single-factor experimental design was employed. A total of 24 male sika deer aged 2–3 years were randomly divided into four groups with six deer per group, including a control group (0% substitution) and three treatment groups fed diets containing 2.8%, 5.6%, and 8.4% fermented rapeseed meal (FRSM), defined as the low (L-FRSM), medium (M-FRSM), and high (H-FRSM) substitution groups, respectively. The feeding trial lasted 63 days, with measurements collected on days 30 and 63. Growth performance, nutrient digestibility, serum biochemical indices, and rectal fecal microbiota were determined. The results showed that the final body weight, total weight gain, and average daily gain L-FRSM were higher in the L-FRSM group than in the control group and other substitution groups (*p* < 0.05), accompanied by a reduced feed conversion ratio (*p* < 0.05). In addition, body height and chest circumference were improved in the L-FRSM group. Regarding nutrient digestibility, the apparent digestibility of neutral detergent fiber and dry matter at day 30, as well as calcium digestibility at day 63 were higher in the L-FRSM group compared to the control and higher-substitution groups (*p* < 0.05). In contrast, crude fat and dry matter digestibility were significantly lower in the H-FRSM group (*p* < 0.05). No statistical differences were observed among treatments in serum biochemical indices related to energy metabolism, protein metabolism, liver function, lipid metabolism, antioxidant capacity, or humoral immunity (*p* > 0.05). Similarly, no significant differences were detected in core microbial composition or α-diversity of rectal fecal microbiota among groups (*p* > 0.05). In conclusion, substituting soybean meal with 2.8% fermented rapeseed meal effectively improves growth performance and nutrient utilization without compromising health status or intestinal microbial stability in sika deer during the pre-antler growth period. The findings provide a scientific basis for optimizing dietary strategies and support the rational application of fermented rapeseed meal in sika deer production.

## Simple Summary

Soybean meal is widely used as a major protein source in sika deer diets. However, concerns about cost stability and sustainability have increased interest in alternative protein ingredients. Fermented rapeseed meal is a potential substitute because fermentation reduces anti-nutritional factors and improves its feeding value. In this study, 24 sika deer were fed diets in which soybean meal was partially replaced with fermented rapeseed meal at different levels for 63 days. We found that a 2.8% replacement level significantly improved growth performance and nutrient digestibility, without negatively affecting animal health or intestinal microbial stability. These results provide practical guidance for optimizing diets in sika deer during the pre-antler growth period.

## 1. Introduction

With the rapid development of modern animal husbandry and the continuous advancement of large-scale production systems, the demand for high-quality protein feed resources has increased substantially. Soybean meal remains the most widely used protein source in animal diets; however, its considerable price fluctuations have become major constraints on feed cost control and the sustainable development of the livestock industry [1,2]. Under increasing pressure from fierce global competition for feed resources and rising feed costs, identifying locally available, cost effective, and nutritionally suitable alternative protein sources have become an important goal for the animal husbandry industry. As a result, exploring and applying innovative protein feed ingredients has attracted growing research interest worldwide.

Rapeseed meal is a by-product of rapeseed oil extraction and is widely available at a relatively low cost. It contains 35–45% crude protein and has a balanced amino acid profile, with a relatively high content of sulfur-containing amino acids, making it a potential protein source for animal diets [3,4]. However, rapeseed meal also contains anti-nutritional factors, such as glucosinolates and phytic acid, which seriously reduce palatability, limit protein digestibility, and may negatively affect animal health. These factors restrict the large-scale direct application of rapeseed meal in animal feeds [5].

In recent years, microbial fermentation has been increasingly applied to improve the nutritional value of agricultural by-products used as feed ingredients. Fermentation using selected microorganisms, including lactic acid bacteria, yeast, and bacillus species, can effectively degrade anti-nutritional factors in rapeseed meal. At the same time, fermentation can increase the levels of crude protein, small peptides, and free amino acids, and generate beneficial substances such as organic acids, enzymes, and vitamins. These changes improve both the nutritional quality and feeding value of rapeseed meal [6,7].

Previous studies have shown that fermented rapeseed meal can be safely used in poultry and ruminants. In yellow-feathered broilers, appropriate supplementation of fermented rapeseed meal did not impair growth performance and was associated with enhanced antioxidant capacity and a healthier intestinal microbial profile [8]. In dairy cows, supplementation of fermented rapeseed meal maintained dry matter intake and milk yield while improving milk quality and rumen fermentation characteristics, and supporting antioxidant and immune functions [9]. In addition, replacing unfermented rapeseed meal with fermented rapeseed meal has been reported to improve rumen fermentation, reduce methane emissions, and optimize milk fatty acid composition [10]. These findings suggest that fermented rapeseed meal has potential as an alternative protein source in ruminant nutrition.

Velvet antler is the primary production objective in sika deer farming and has long been valued in traditional medicine [11,12,13], and in recent years, it has been proven to have some benefits in health, such as anticancer effects [14]. Antler size is strongly influenced by the dietary nutrient supply and nutritional management during the months of antler growth for sika deer (May–September). The pre-antler period (typically May–June) is one of several key stages in the annual cycle that significantly supports overall antler development, when protein availability and nutrient utilization are critical. Currently, the diets of farmed sika deer are predominantly based on corn and soybean meal, resulting in a limited diversity of protein sources and relatively high feed costs. During the pre-antler period, stage, partial replacement of soybean meal with alternative protein sources, such as fermented rapeseed meal, may represent a cost-effective strategy to diversify dietary protein sources and improve nutrient availability.

We hypothesized that partial substitution of soybean meal with fermented rapeseed meal during the pre-antler period would maintain or improve nutrients utilization efficiency without compromising physiological status in sika deer. To test this hypothesis, we evaluated the effects of replacing soybean meal with fermented rapeseed meal on growth performance, nutrient digestibility, serum biochemical parameters, and rectal fecal microbiota in sika deer during the pre-antler period. The findings of this study aim to provide practical guidance for diet formulation and support the rational use of fermented rapeseed meal in sika deer farming systems.

## 2. Materials and Methods

### 2.1. Experimental Design and Feeding Management

Twenty-four healthy male sika deer aged 2–3 years, with an average body weight of 98.95 ± 0.55 kg and similar antler casting times, were selected for this experiment. The experiment was conducted at the Sika Deer Breeding Farm in Zuojia, Jilin City, Jilin Province, China. The fermented rapeseed meal (FRSM) used was purchased from Hubei Bangzhide Animal Husbandry Technology Co., Ltd., Wuhan, China), and the nutritional components of fermented rapeseed meal are shown in Table 1. All animal experiments were approved by the Animal Ethics Committee of the Chinese Academy of Agricultural Sciences and were conducted in accordance with the guidelines for animal experiments formulated by the National Institute of Animal Health (NO. ISAPSAEC-2022-61D).

The 24 sika deer were randomly allocated to four groups with six replicates per group and one deer per replicate. Each group was housed in an independent pen, resulting in a total of four pens. Throughout the experimental period, ambient temperature ranged from 10 °C to 28 °C, and pens were regularly cleaned and disinfected to maintain hygienic conditions.

All deer were fed a basal diet consisting of corn silage, peanut vines, and concentrate (The ratio of roughage to concentrate was 30:70 on a dry matter basis). The animals were randomly assigned to one of the following four dietary treatments (Table 2): control group (CON) no fermented rapeseed meal; a low-level FRSM group (L-FRSM) receiving 2.8% FRSM; a medium-level FRSM group (M-FRSM) receiving 5.6% FRSM; and a high-level FRSM (H-FRSM) receiving 8.4% FRSM. The control diet was formulated according to the Chinese national standard GB/T 41190-2021 [15] “*Nutrient Requirements of Deer*,” to meet the nutritional requirements of sika deer during the antler growth period. All experimental diets were designed to be isonitrogenous and isocaloric across treatments. Deer were fed three times a day at fixed times (4:00, 10:00, and 16:00), with ad libitum access to drinking water. Feed intake was recorded daily. The experiment lasted for 70 days, including a 1-week adaptation period.

### 2.2. Determination of Body Weight, Body Size, and Feed Intake

All sika deer were fasted and weighed before morning feeding on the 1st day and the final day of the experiment to record initial weight (IW) and final weight (FW), which were used for calculating total weight gain (TWG) and average daily weight gain (ADWG).

TWG = FW − IW

ADWG = TWG/63

Daily feed intake was recorded throughout the experiment for the calculation of average daily feed intake (ADFI) and feed-to-weight ratio (F/W). Main body size indices of all sika deer were measured with a measuring stick and a tape measure when the deer stood naturally on flat ground; each index was determined in triplicate and the mean value was adopted, including withers height (WH), body length (BL), heart girth (HG), cannon bone circumference (CBC), and hip height (HH), all expressed in centimeters (cm).

### 2.3. Sample Collection and Chemical Analysis of Diet and Feces

Prior to the start of the experiment, approximately 500 g of thoroughly mixed finished diet samples were collected from each treatment group. Samples were dried in a drying oven at 65 °C to constant weight. The dried samples were then ground and passed through 40-mesh and 100-mesh sieves. The processed samples were sealed in plastic bags and stored at −20 °C for subsequent analysis of nutritional components.

A digestive trial was conducted monthly using the partial fecal collection method with acid-insoluble ash (AIA) as an internal indigestible marker. Fecal samples were collected continuously for three consecutive days. Pens were cleaned before morning feeding each day, and fresh feces excreted by each sika deer within 24 h were collected. After removing contaminants (e.g., hair), feces were weighed, thoroughly mixed, and subsampled at 10% of the total fecal output. Dilute sulfuric acid was added to the subsamples for nitrogen fixation. Fecal samples were then ground, sieved, and crude protein content was determined using the Dumas combustion method (C.Gerhardt, Guangzhou, China; Königswinter, Germany). Crude protein was calculated from total nitrogen according to ISO 16634-1:2008 [16]. All fecal samples were stored at −20 °C until analysis. After completing the digestive trial, frozen fecal samples were thawed, homogenized, and initially dried at 85 °C for 2 h for sterilization, followed by further drying at 65 °C to constant weight. After cooling, moisture content was determined. The dried fecal samples were ground, passed through a 40-mesh sieve, sealed in plastic bags, and stored at −20 °C for subsequent analysis. Total fecal output was estimated using acid-insoluble ash as an internal marker [17]. The contents of dry matter (DM), ether extract (EE), calcium (Ca), phosphorus (P), neutral detergent fiber (NDF), and acid detergent fiber (ADF) were determined according to the methods of AOAC [18]. The gross energy (GE) content was determined using an isoperibol bomb calorimeter (IKA C2000; IKA, Staufer, Germany).

### 2.4. Serum Collection and Biochemical Analysis

On the final day of the experiment and prior to morning feeding, 15 mL of blood was collected from the jugular vein of each sika deer using vacuum blood collection tubes containing a coagulant. Samples were allowed to clot at −4 °C for 60 min and then centrifuged at 4500 r/min for 15 min. The serum was carefully collected, aliquoted into 1.5 mL tubes (1000 μL per tube), snap-frozen in liquid nitrogen, and stored at −80 °C until analysis.

Serum biochemical indices were measured using a WetLab-E automatic biochemical analyzer, with commercial assay kits (Biosino Biotechnology and Science Co., Ltd., Beijing, China) following the manufacturer’s instructions. The measured indices included glucose (GLU), total Protein (TP), albumin (ALB), alkaline phosphatase (ALP), aspartate aminotransferase (AST), alanine aminotransferase (ALT), urea nitrogen (Urea), triglyceride (TG), total cholesterol (CHO), high-density lipoprotein cholesterol (HDL-C), and low-density lipoprotein cholesterol (LDL-C). Serum immunoglobulin A (IgA), immunoglobulin M (IgM), and immunoglobulin G (IgG) concentrations were determined by enzyme-linked immunosorbent assay (ELISA) using commercial kits (Jiangsu Sophia Biotechnology Co., Ltd., Yancheng, China). Antioxidant-related indices, including catalase (CAT), glutathione Peroxidase (GSH-Px), total superoxide dismutase (T-SOD), total antioxidant capacity (T-AOC), and malondialdehyde (MDA), were measured suing assay kits from Nanjing Jiancheng Bioengineering Institute (Nanjing, China).

### 2.5. Rectal Fecal Sample Collection for Microbiome Analysis

Prior to morning feeding on the final day of the experiment, fresh fecal samples (approximately 5 g) were collected directly from the rectum of each sika deer using sterile disposable gloves. Samples were placed into sterilized 5 mL cryogenic tubes, snap-frozen in liquid nitrogen, transported to the laboratory, and stored at −80 °C until microbial genomic DNA extraction.

### 2.6. DNA Extraction, PCR Amplification, and Sequencing of Gastrointestinal Microbiota

Total genomic DNA samples were extracted using the MagBeads FastDNA Kit for Soil (116564384) (MP Biomedicals, CA, USA), following the manufacturer’s instructions, and stored at −20 °C until further analysis. The quantity and quality of extracted DNAs were measured using a NanoDrop NC2000 spectrophotometer (Thermo Fisher Scientific, Waltham, MA, USA) and agarose gel electrophoresis, respectively. PCR amplification of the bacterial 16S rRNA genes V3–V4 region was performed using the forward primer 338F (5′-ACTCCTACGGGAGGCAGCA-3′) and the reverse primer 806R (5′-GGACTACHVGGGTWTCTAAT-3′). High-throughput sequencing library construction and Illumina MiSeq sequencing were performed by Personal Biotechnology Co., Ltd. (Personalbio, Shanghai, China). Bioinformatic analysis of the microbiome was conducted using QIIME2 2024.05. Quality filtering was performed on the raw paired-end sequences to remove low-quality reads, reads containing N bases, and adapter-contaminated sequences. The obtained sequences were clustered into operational taxonomic units (OTUs) at a 97% similarity level, and the representative sequences were taxonomically assigned from phylum to species level. Representative sequences of each OTU were taxonomically annotated and classified into the hierarchical levels of phylum, class, order, family, and genus. QIIME2 2024.05 software was used to calculate the microbial community richness and evenness indices for each sample, including the Observed species, Chao1 index (community richness), Shannon index and Simpson index. Based on the Unweighted UniFrac distance matrix, principal coordinates analysis (PCoA) was performed to visualize the differences in microbial community structure among experimental groups. The composition of the microbial community in each group was further analyzed at the taxonomic levels of phylum, class, order, family, and genus.

### 2.7. Statistical Analysis

Statistical analyses were performed using SAS 9.4 software (SAS Institute, Inc., Cary, NC, USA). Growth performance, body size indices, nutrient digestibility, and serum biochemical parameters were analyzed using one-way analysis of variance (ANOVA), with dietary treatment as the fixed effect. When significant differences were detected, Duncan’s multiple range test was applied for post hoc comparisons. Microbial α-diversity indices were analyzed using one-way ANOVA. All data were expressed as mean ± standard error (Mean ± SE). Differences were considered statistically significant at *p* < 0.05.

## 3. Results

### 3.1. Effects of Fermented Rapeseed Meal Substitution at Different Ratios on Growth Performance of Sika Deer During the Pre-Antler Growth Period

As shown in Table 3, at the end of the experimental period, FW, TWG, and ADWG differed significantly among treatments (*p* < 0.05). Specifically, FW, TWG, and ADWG in the L-FRSM group were significantly higher than those of the control, medium-level substitution, and high-level substitution groups (*p* < 0.05). The body FW of deer in the L-FRSM group was approximately 1.87 kg greater than that of the control group, and the ADWG reaching 220.63 g/d, which was significantly higher than the 187.83 g/d observed in the control group. In contrast, no significant differences in FW, TWG, and ADWG were detected between the medium- and high-level substitution groups and the control group, with both substitution groups showing slightly lower values than the control.

In terms of feed intake, ADFI did not differ significantly among groups, indicating that fermented rapeseed meal substitution did not affect feed consumption in sika deer. Regarding feed utilization efficiency, significant differences were observed in the F/W (*p* < 0.05). The L-FRSM group exhibited the lowest F/W ratio (13.44%), which was significantly lower than that of the CON group and the other fermented rapeseed meal-substituted groups. This result indicated that the sika deer in the L-FRSM group achieved the highest efficiency in converting feed into body weight gain. Conversely, the medium- and high-level substitution groups showed higher F/W ratios, reflecting relatively lower feed conversion efficiency compared with the L-FRSM group.

### 3.2. Effects of Different Proportions of Fermented Rapeseed Meal Substitution on Body Size Indicators of Sika Deer During the Pre-Antler Growth Period

As shown in Table 4, no significant differences were observed in initial body height, diagonal body length, chest circumference, CBC, or HH among all groups at the start of the experiment (*p* > 0.05). Following the experimental period, fermented rapeseed meal supplementation exerted selective effects on specific final body size indices of sika deer. Final body height in the L-FRSM group was significantly higher than that in the control, medium-level substitution, and high-level substitution group (*p* < 0.05). Similarly, the final chest circumference of deer in the L-FRSM group was significantly greater than that of all other groups (*p* < 0.05). In contrast, no significant differences were detected in final diagonal body length, CBC, or HH among treatment groups (*p* > 0.05).

### 3.3. Effects of Different Proportions of Fermented Rapeseed Meal Substitution Levels on Nutrient Digestibility in Sika Deer During the Pre-Antler Growth Period

Apparent nutrient digestibility was determined on day 30 and day 60 using the partial collection method, and the results are presented in Table 5. Results from day 30 showed that the apparent digestibility of NDF in the L-FRSM group was significantly higher than that in the other three groups (*p* < 0.05). No significant differences were observed in the digestibility of CP, EE, ADF, Ca, P, or GE among treatment groups (*p* > 0.05) after 30 days of feeding. Results from day 60 (Table 5) indicated that the L-FRSM group showed a trend toward higher CP digestibility, and its Ca digestibility was significantly higher than that of the medium- and high-level substitution groups (*p* < 0.05). In contrast, the H-FRSM group exhibited a significant negative effect on nutrient digestibility, with EE and DM digestibility being significantly or lower than those of all other groups (*p* < 0.05). No significant differences were detected in the apparent digestibility of CP, NDF, ADF, P, or GE among treatment groups during this period (*p* > 0.05).

### 3.4. Effects of Different Proportions of Fermented Rapeseed Meal Substitution on Serum Biochemical Indices of Sika Deer During the Pre-Antler Growth Period

As shown in Table 6, comparison of serum indices among the CON, L-FRSM, M-FRSM, and H-FRSM groups revealed no significant changes in GLU, TP, or ALB (*p* > 0.05). Serum levels of ALP, AST, and ALT, commonly used as indicators of liver function, did not differ significantly among groups (*p* > 0.05). Antioxidant-related indices, including CAT, GSH-PX, T-SOD, T-AOC, and the lipid peroxidation product MDA, remained comparable across all treatment groups (*p* > 0.05). Similarly, lipid metabolism indicators such as TG, CHO, HDL-C, and LDL-C, showed no significant difference among groups (*p* > 0.05). Immune function indicators, including IgA, IgM, IgG, and BUN, an indicator of renal function, also did not differ significantly among groups with different substitution ratios (*p* > 0.05). In summary, all the determined serum indices did not show significant differences among the four groups, suggesting that fermented rapeseed meal substitution did not adversely affect the overall health status of the animals.

### 3.5. Effects of Different Proportions of Fermented Rapeseed Meal Substitution on Rectal Fecal Microflora of Sika Deer During the Pre-Antler Growth Period

**Figure 1:**
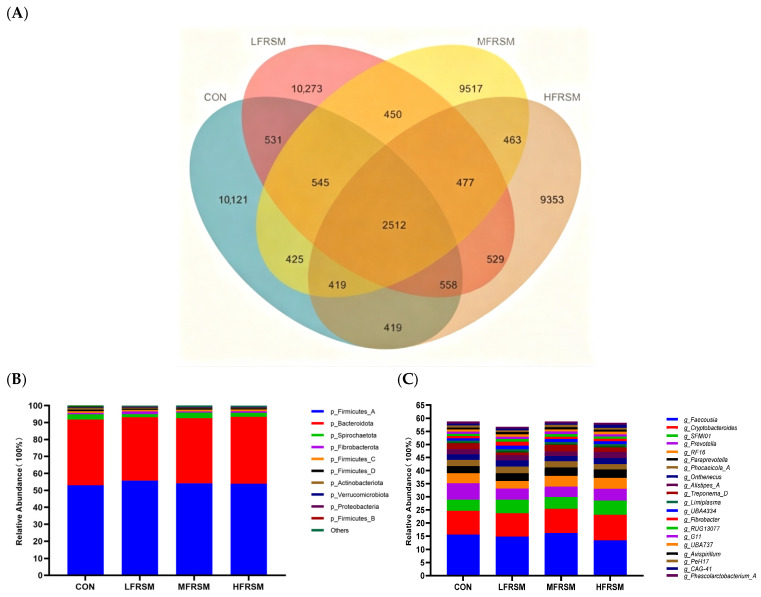
Overall structure of the rectal fecal microbiota in sika deer during the pre-antler growth period. (**A**) Venn diagram illustrating shared and unique operational taxonomic units (OTUs) among the four groups. (**B**) Relative abundances of dominant bacterial taxa at the phylum level. (**C**) Relative abundances of dominant bacterial taxa at the genus levels.

The overall structure of the intestinal microflora of sika deer was visualized using a Venn diagram (Figure 1A) and taxonomic composition bar charts at the phylum and genus levels (Figure 1B, Figure 1C, respectively). The Venn diagram showed that at a 97% similarity threshold, a total of 46,592 operational taxonomic units (OTUs) were identified, with 2512 core operational taxonomic units (OTUs) shared among the four groups. The number of unique OUTs was 10,121 in the CON group, 10,273 in the L-FRSM group, 9517 in the M-FRSM group, and 9353 in the H-FRSM group. At the phylum level (Figure 1B), the microbial community composition was similar across groups, with *Firmicutes* and *Bacteroidota* being the dominant phyla, together accounting for more than 85% the total relative abundance. At the genus level (Figure 1C), *Faecousia*, *Cryptobacteroides*, *SFMI01*, and *Prevotella* were the dominant genera shared among all groups.

As shown in Table 7, substitution of soybean meal with fermented rapeseed meal had no significant effect on α-diversity indices, including Chao1 index, Shannon index, and Observed_species index), among the experimental groups (*p* > 0.05). Consistently, no significant differences in microbial richness or diversity were observed between any treatment group and the control group.

**Figure 2:**
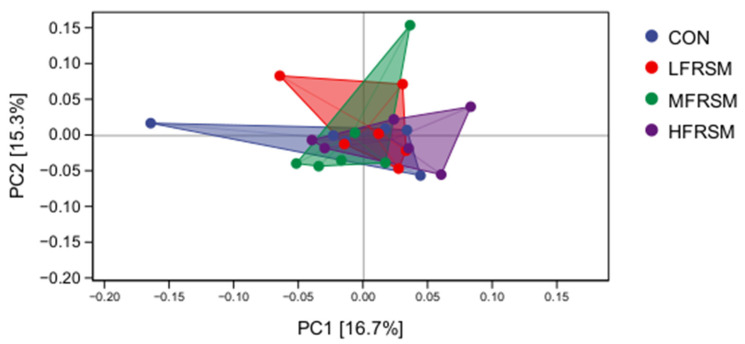
Principal coordinate analysis (PCoA) plot of rectal fecal microbiota community structure based on Weighted Unifrac distance among different treatment groups.

Principal Coordinate Analysis (PCoA) based on Weighted Unifrac distance showed a certain degree of clustering within groups; however, substantial overlap was observed among groups (Figure 2). The first principal coordinate (PC1) and the second principal coordinate (PC2) explained 16.7% and 15.3% of the total variation in microbial community composition among samples, respectively.

## 4. Discussion

The present study evaluated the effects of replacing soybean meal with fermented rapeseed meal at different proportions (2.8%, 5.6%, and 8.4%) on growth performance, body size, nutrient digestibility, serum biochemistry, and intestinal microbiota in sika deer during the pre-antler growth period. Overall, the results indicate that a moderate substitution level (2.8%) improved growth performance and nutrient utilization without adversely affecting health status or intestinal microbial stability.

### 4.1. Growth Performance

The results indicated that low-level replacement of soybean meal with fermented rapeseed meal (L-FRSM group) significantly improves growth performance in sika deer during the early antler-growing period, as manifested by the significant increase in final body weight, TWG, and average daily gain, along with a decrease in feed-to-weight ratio. In contrast, medium- and high-level replacements did not further enhance growth performance and showed a slight inhibitory trend. These findings are partially consistent with previous studies in poultry and ruminants. For example, appropriate addition of fermented rapeseed meal has been reported to improve growth performance and egg quality in laying hens [19], and to improve their growth performance and meat quality in yellow-feathered broilers, whereas excessive inclusion decreased growth performance [8]. Similarly, in dairy cows, partial replacement of soybean meal with fermented rapeseed meal improved milk production without affecting dry matter digestibility [10].

The positive effects observed at low-level substitution may be attributed to fermentation-induced degradation of anti-nutritional factors such as glucosinolates and phytic acid in rapeseed meal [20], which reduces intestinal irritation. In addition, fermentation increases the contents of crude protein, small peptides, and free amino acids in the diet, thereby improving dietary nutritional value by enhancing protein utilization efficiency, leading to improved growth performance in the L-FRSM group. At medium- and high-level replacements, residual anti-nutritional factors in fermented rapeseed meal may accumulate and reduce feed conversion efficiency, ultimately limiting growth performance [21]. In the present study, the estimated dietary glucosinolate levels (0.032–0.096 μmol/g) were far below the concentrations reported to impair feed intake or metabolism in ruminants (~1.5–4.2 μmol/g diet) [22,23]. Similarly, the calculated dietary concentrations of isothiocyanates (3.11–9.32 mg/kg), oxazolidinethione (7.36–22.09 mg/kg), tannins (0.00017–0.00050%), and phytic acid (≤0.0028–0.0084%) were relatively low [24,25]. However, although fermentation substantially reduces these anti-nutritional factors, increasing the inclusion level of fermented rapeseed meal may elevate the cumulative intake of residual compounds. Glucosinolates and their hydrolysis products, such as isothiocyanates and oxazolidinethiones, have been reported to affect metabolic processes and endocrine regulation associated with growth in animals [22,23]. Therefore, the reduced FW gain and height gain observed in the high-replacement group may be partly associated with the cumulative effects of residual anti-nutritional compounds at higher dietary inclusion levels.

Body size traits are important phenotypic indicators reflecting animal growth and development. Among them, body height and chest circumference are mainly associated with skeletal growth and muscle development, whereas diagonal body length and cannon bone circumference reflect body fullness and limb robustness [26]. Chest circumference is generally considered the body measurement most strongly correlated with body weight, followed by diagonal body length and body height [27]. In this study, animals in the L-FRSM group exhibited significantly higher final body height and chest circumference than those in other groups, suggesting enhanced skeletal and muscle. This improvement is likely associated with the higher nutrient digestibility observed in this group. In contrast, medium- and high-level replacement did not confer advantages in body size traits, highlighting the importance of appropriate inclusion levels.

### 4.2. Nutrient Digestibility

Nutrient digestibility is the core index for evaluating feed nutritional value, as it directly determines nutrient absorption and utilization efficiency, thereby affecting animal growth performance [28,29,30,31]. On day 30 after feeding, the L-FRSM group showed significantly higher apparent digestibility of NDF and DM compared with other groups, along with a trend toward increased CP digestibility, while no significant differences were observed for EE. As a main component of structural carbohydrates, NDF plays a critical role in ruminant performance. The increased NDF and DM digestibility at the low-level replacement may result from fermentation-induced improvements in physical and chemical properties of the diet, including elevated content of small peptides and free amino acids in the diets [7], which are more readily absorbed and utilized by animals. These changes may provide an important nutritional basis for the trend toward increased CP digestibility and the improved growth performance observed in the L-FRSM group.

On day 60 post feeding, the L-FRSM group tended to maintain higher CP digestibility and showed a similar trend toward increased Ca digestibility than the medium- and high-level replacement groups, whereas the H-FRSM group exhibited a significant decrease in EE and DM digestibility. Ca is essential for bone calcification and antler development, and its digestibility and absorption efficiency directly affects antler growth [32,33]. At a high-level substitution level, residual phytic acid in fermented rapeseed meal may form insoluble complexes with Ca, thereby reducing calcium digestibility [34]. The decrease in dry matter digestibility and fat digestibility observed in the H-FRSM group likely reflects impaired intestinal digestive function in sika deer caused by excessive inclusion of fermented rapeseed meal. Although fermentation substantially reduces these anti-nutritional factors, increasing the inclusion level of fermented rapeseed meal may elevate the cumulative intake of residual compounds. Glucosinolates can be hydrolyzed to biologically active products such as isothiocyanates and oxazolidinethiones, which may interfere with nutrient metabolism and physiological functions in animals [22,23], while tannins and phytic acid can bind proteins and minerals, thereby reducing nutrient availability and digestive efficiency [24,25]. In addition, the increased ADF content in the H-FRSM diet may further limit nutrient accessibility and digestibility in ruminants [35], the higher ADF content in the H-FRSM group may have contributed to the reduced digestibility of DM, Ca, and EE. ADF reflects the relatively indigestible cell wall fraction, particularly cellulose and lignin, which can limit microbial degradation in the rumen and thereby reduce nutrient availability [36,37]. Together, these factors may partially explain the reduced nutrient digestibility observed at higher substitution levels. Indeed, it is reported that the fermented rapeseed meal in the total mixed ration of dairy cows should not exceed 12.11%, beyond which negative effects on production performance, serum biochemical indicators, and nutrient digestibility may occur. Similarly, excessive inclusion of fermented rapeseed meal in the diet of meat sheep resulted in abnormal decrease in feed intake and growth rate, presumably due to different fermentation procedures [38].

These findings suggest that the effects of fermented rapeseed meal depend on the level of substitution. In the present study, low-level replacement significantly increased NDF and DM digestibility on day 30 and showed a tendency toward higher CP and Ca digestibility, which may be attributed to fermentation-induced improvements in the physical and chemical properties of the diet. In contrast, excessive substitution (H-FRSM) resulted in significantly reduced DM and EE digestibility on day 60, likely due to the accumulation of residual anti-nutritional factors and higher content of ADF associated with fermented rapeseed meal. Overall, these findings indicate that moderate inclusion of fermented rapeseed meal can enhance nutrient utilization, whereas higher substitution levels may impair digestive efficiency in sika deer.

### 4.3. Serum Biochemical Indicators

Serum biochemical indices provide important information on physiological status, metabolic function, and overall health. Serum GLU reflects energy availability and metabolism in animals [39], while ALB and TP play crucial regulatory roles in binding hormones, fatty acids, and bilirubin, regulating vascular permeability, and maintaining osmotic pressure [40]. BUN is an end product of protein metabolism, and elevated levels may indicate impaired renal function. In this study, no significant differences were observed in GLU, TP, ALB, or BUN among groups, indicating that fermented rapeseed meal substitution did not adversely affect energy or protein metabolism. ALP, AST, and ALT are commonly used indicators of bone metabolism and liver function [40,41,42,43]. No significant changes in these enzymes were observed, suggesting that fermented rapeseed meal did not impair bone development or liver function. CAT,, GSH-PX, and T-AOC play key roles in maintaining redox balance, whereas MDA reflects lipid peroxidation and oxidative damage [44,45]. These indicators were measured to evaluate the oxidative stress status of sika deer. No significant differences in these indices were observed, indicating that fermented rapeseed meal substitution did not alter oxidative stress status in sika deer. Similarly, lipid metabolism indicators (TG, CHO, HDL-C, and LDL-C) also remained unchanged, indicating minimal effects on lipid metabolism. Furthermore, no significant differences were found in IgA, IgM, or IgG levels, suggesting that humoral immune function was not affected.

These results indicate that replacing soybean meal with fermented rapeseed meal at the tested levels (0–8.4%) did not affect liver and kidney function, antioxidant capacity, and immune function of sika deer during the pre-antler stage. It indicates that the replacement with fermented rapeseed meal within the ratio range has high safety and will not cause adverse effects on the metabolic function.

### 4.4. Intestinal Microbiota

The gut microbiota is directly associated with nutrient digestion and absorption, immune regulation, and host growth [46,47]. In this study, 16S rRNA gene sequencing was employed to evaluate the effects of fermented rapeseed meal substitution on rectal fecal microbiota. Low-level substitution increased OTU richness, whereas high-level substitution reduced it, suggesting that the level of fermented rapeseed meal affected the establishment of intestinal microflora in sika deer during the pre-antler-growing period. However, core composition (at both phylum and genus level) and α-diversity indices were not significantly altered, indicating overall microbial stability. These observations were confirmed by principal coordinate analysis (PCoA) based on Weighted Unifrac distance, which revealed no significant differences in the overall community structure of the intestinal microbiota among all groups. In addition, although some unique OTUs were identified in individual groups, functional prediction based on KEGG pathways showed no significant differences (Figure S1). This may be attributed to the functional redundancy of intestinal microbial communities, in which taxonomically distinct microorganisms often perform similar metabolic functions. Moreover, unique OTUs are typically present at low abundance and therefore contribute minimally to the overall functional potential of the microbiota. Overall, fermented rapeseed meal substitution at levels up to 8.4% did not disrupt intestinal microbial structure or diversity, indicating good microecological safety.

However, we acknowledge an important limitation of the present study. In ruminants, dietary protein digestion and microbial fermentation primarily occur in the rumen, which represents the central metabolic compartment where FRSM substitution is most likely to exert its regulatory effects on nutrient metabolism and microbial community structure. Although rectal fecal microbiota analysis provides valuable information regarding hindgut microbial status, it cannot serve as a direct proxy for rumen microbiota and therefore does not fully capture the effects of FRSM at the primary site of gastrointestinal fermentation. Future studies incorporating rumen microbiota profiling and rumen fermentation parameters will be necessary to more comprehensively elucidate the mechanisms underlying FRSM substitution in sika deer.

## 5. Conclusions

Under the conditions of this experiment, replacing soybean meal with 2.8% fermented rapeseed meal was identified as the optimal dietary proportion for sika deer during the pre-antler-growing period. This substitution level significantly improved growth performance and certain aspects of nutrient digestion while maintaining physiological health and intestinal microbial stability in sika deer.

These findings provide a scientific basis for the rational utilization of fermented rapeseed meal as an alternative protein source in sika deer production and support its application for cost reduction and sustainable industry development.

## Figures and Tables

**Table 1:** Nutritional levels of fermented rapeseed meal (%, air-dried basis).

Nutrient Levels %	Content %	Anti-Nutritional Factors	Content
DM	90.46	Glucosinolates (Sinigrin equivalent)	1.14 μmol/g
CP	42.13	Isothiocyanates	111 mg/kg
EE	4.38	Oxazolidinethione	263 mg/kg
ASh	6.92	Tannic acid	0.006%
NDF	29.52	Phytic acid	≤0.1%
ADF	19.94		
Ca	1.54		
P	0.65		

CP, crude protein; DM, dry matter; EE, ether extract; Ca, calcium; P, phosphorus; NDF, neutral detergent fiber; ADF, acid detergent fiber.

**Table 2:** Ingredients and nutrient composition of experimental diets (%, air-dried basis).

Parameter	CON	L-FRSM	M-FRSM	H-FRSM
Ingredients (%)				
corn silage	17.00	17.00	17.00	17.00
peanut vines	13.00	13.00	13.00	13.00
Corn	23.10	22.40	21.70	21.00
Soybean meal	19.60	16.80	14.00	11.20
fermented rapeseed meal	0.00	2.80	5.60	8.40
corn germ meal	7.28	7.94	8.40	8.40
corn germ cake	4.62	4.65	4.90	5.60
Alfalfa (CP 17%)	9.80	9.80	9.80	9.80
Calcium bicarbonate·2H_2_O	1.61	1.51	1.47	1.47
molasses	2.10	2.10	2.10	2.10
NaHCO_3_	0.70	0.70	0.70	0.70
NaCl	0.21	0.21	0.21	0.21
Premix ^1^	0.98	1.09	1.12	1.12
Total	100.00	100.00	100.00	100.00
Nutrient levels (% DM)				
GE (MJ/kg DM)	17.05	17.20	17.27	17.32
DM	78.16	78.74	79.08	79.12
CP	19.46	19.75	19.67	19.73
EE	2.74	2.79	2.88	2.80
ASh	8.58	8.20	8.17	8.19
NDF	29.99	29.70	29.91	30.49
ADF	18.35	17.83	17.73	18.96
Ca	1.05	0.98	0.99	1.02
P	0.46	0.46	0.49	0.53

CP, crude protein; DM, dry matter; GE, gross energy; EE, ether extract; Ca, calcium; P, phosphorus; NDF, neutral detergent fiber; ADF, acid detergent fiber. ^1^ One kilogram of premix contained the following: VA, 12,000 U; VB_1_, 0.0020 mg; VB_2_, 0.015 mg;VD_3_, 2500 IU; VE, 30 IU; VK_3_, 0.005 mg; folic acid, 0.0004 mg; niacin, 0.030 mg; Fe, 0.95 mg; Mn, 0.78 mg; zinc, 0.65 mg; selenium, 0.56 mg; copper, 10 mg; iodine, 0.35 mg.

**Table 3:** Effects of different substitution levels of fermented rapeseed meal on growth performance of sika deer during the pre-antler growth period (mean ± SE, *n* = 6).

Items	IW, kg	FW, kg	TWG, kg	ADWG, g/d	ADFI, kg/d	F/W
CON	99.00 ± 0.18	110.83 ± 0.36 ^b^	11.83 ± 0.36 ^b^	187.83 ± 5.69 ^b^	2.93 ± 0.03	15.70 ± 0.58 ^b^
L-FRSM	98.80 ± 0.27	112.70 ± 0.36 ^a^	13.90 ± 0.27 ^a^	220.63 ± 4.36 ^a^	2.96 ± 0.01	13.44 ± 0.25 ^a^
M-FRSM	99.10 ± 0.24	110.62 ± 0.29 ^b^	11.52 ± 0.39 ^b^	182.80 ± 6.15 ^b^	2.97 ± 0.02	16.36 ± 0.60 ^b^
H-FRSM	98.90 ± 0.27	110.28 ± 0.28 ^b^	11.38 ± 0.33 ^b^	180.69 ± 5.17 ^b^	2.97 ± 0.02	16.51 ± 0.40 ^b^
*p*-values	0.836	<0.05	<0.05	<0.05	0.427	<0.05

IW, initial weight; FW, final weight; TWG, total weight gain; ADWG, average daily weight gain; ADFI, average daily feed intake; F/W, weight gain to feed ratio. CON, Control group; L-FRSM, Low-level fermented rapeseed meal group; M-FRSM, Medium-level fermented rapeseed meal group; H-FRSM, High-level fermented rapeseed meal group. Values in the same column with different superscripts differ significantly (*p* < 0.05).

**Table 4:** Effects of different substitution levels of fermented rapeseed meal on body size indices in sika deer during the pre-antler growth period (mean ± SE, *n* = 6).

Item	IWH, cm	FWH, cm	IBL, cm	FBL, cm	ICBC, cm	FCBC, cm	IHG, cm	FHG, cm	IHH, cm	FHH, cm
CON	96.01 ± 0.17	101.93 ± 0.20 ^b^	122.13 ± 0.32	125.68 ± 0.30	14.83 ± 0.92	14.17 ± 0.13	126.00 ± 0.14	132.53 ± 0.21 ^b^	106.00 ± 0.14	116.38 ± 1.11
L-FRSM	95.99 ± 0.29	102.80 ± 0.26 ^a^	122.07 ± 0.56	126.20 ± 0.50	14.95 ± 1.66	14.30 ± 0.20	125.95 ± 0.21	133.48 ± 0.21 ^a^	105.93 ± 0.23	117.92 ± 1.84
M-FRSM	96.19 ± 0.25	101.95 ± 0.21 ^b^	122.33 ± 0.40	125.77 ± 0.34	14.78 ± 0.41	14.20 ± 0.13	126.10 ± 0.16	132.45 ± 0.18 ^b^	106.12 ± 0.17	116.23 ± 0.55
H-FRSM	95.97 ± 0.28	101.67 ± 0.24 ^b^	121.88 ± 0.57	125.28 ± 0.52	14.63 ± 1.10	14.03 ± 0.17	125.95 ± 0.17	132.18 ± 0.15 ^b^	105.95 ± 0.17	115.92 ± 0.96
*p*-values	0.916	<0.05	0.929	0.522	0.935	0.697	0.920	<0.05	0.890	0.658

IWH, initial withers height; IBL, initial body length; IHG, initial heart girth; ICBC, initial cannon bone circumference; IHH, initial hip height; FWH, final withers height; FBL, final body length; FHG, final heart girth; FCBC, final cannon bone circumference; FHH, final hip height. Values in the same column with different superscripts differ significantly (*p* < 0.05).

**Table 5:** Effects of different substitution levels of fermented rapeseed meal on apparent nutrient digestibility in sika deer during the pre-antler growth period (mean ± SE, *n* = 6).

Items	CON	L-FRSM	M-FRSM	H-FRSM	*p*-Values
30 d nutrient apparent digestibility (%) ^1^					
GE	69.36 ± 0.65	70.66 ± 0.20	68.56 ± 0.98	69.60 ± 1.08	0.347
CP	68.74 ± 0.46	69.92 ± 0.41	68.14 ± 0.69	67.95 ± 0.64	0.072
EE	86.67 ± 0.57	86.69 ± 0.45	86.49 ± 0.41	85.44 ± 0.83	0.391
NDF	44.29 ± 1.14 ^b^	47.97 ± 0.57 ^a^	44.18 ± 0.68 ^b^	43.32 ± 1.52 ^b^	0.015
ADF	49.58 ± 0.98	51.00 ± 0.64	49.49 ± 0.51	49.04 ± 1.23	0.446
DM	92.60 ± 0.14 ^ab^	93.26 ± 0.21 ^a^	92.94 ± 0.25 ^ab^	92.07 ± 0.46 ^b^	0.038
Ca	30.85 ± 0.93	31.75 ± 0.51	30.35 ± 2.63	29.00 ± 1.32	0.661
P	30.57 ± 0.51	32.40 ± 2.79	30.10 ± 0.58	29.84 ± 0.56	0.612
60 d nutrient apparent digestibility (%) ^2^					
GE	70.07 ± 0.32	70.55 ± 0.61	70.69 ± 0.55	69.53 ± 0.70	0.473
CP	71.54 ± 0.32	72.05 ± 0.35	71.53 ± 0.57	70.46 ± 0.47	0.088
EE	87.17 ± 0.20 ^a^	87.65 ± 0.87 ^a^	87.14 ± 0.80 ^a^	83.22 ± 0.74 ^b^	<0.05
NDF	44.86 ± 0.65	47.18 ± 1.30	44.70 ± 1.16	44.01 ± 1.00	0.178
ADF	45.95 ± 0.53	46.19 ± 1.29	45.60 ± 1.69	45.03 ± 1.04	0.915
DM	90.83 ± 0.19 ^a^	90.74 ± 0.23 ^a^	90.56 ± 0.20 ^a^	89.42 ± 0.30 ^b^	<0.05
Ca	29.83 ± 0.57 ^ab^	31.32 ± 0.17 ^a^	28.46 ± 0.48 ^bc^	27.28 ± 0.79 ^c^	<0.05
P	34.55 ± 0.61	34.67 ± 0.30	33.93 ± 0.39	33.85 ± 0.55	0.525

DM, dry matter; CP, crude protein; EE, ether extract; Ca, calcium; P, phosphorus; NDF, neutral detergent fiber; ADF, acid detergent fiber. ^1^ Determination time point of nutrient apparent digestibility on day 30 of the feeding trial; ^2^ Determination time point of nutrient apparent digestibility on day 60 of the feeding trial. Values in the same row with different superscripts differ significantly (*p* < 0.05).

**Table 6:** Effects of fermented rapeseed meal substitution at different levels on serum biochemical indices in sika deer during the pre-antler growth period (mean ± SE, *n* = 6).

Items	CON	L-FRSM	M-FRSM	H-FRSM	*p*-Values
GLU	8.72 ± 0.21	8.86 ± 0.01	8.60 ± 0.13	8.45 ± 0.12	0.209
TP	67.55 ± 0.43	67.83 ± 0.25	66.80 ± 0.40	66.57 ± 0.40	0.084
ALB	26.46 ± 0.22	26.88 ± 0.24	26.19 ± 0.39	26.02 ± 0.21	0.169
ALP	144.33 ± 0.51	143.33 ± 0.65	146.49 ± 0.51	147.25 ± 1.21	0.060
AST	50.08 ± 1.66	48.90 ± 1.05	51.00 ± 1.51	52.85 ± 1.11	0.243
ALT	36.58 ± 1.52	36.29 ± 1.92	38.81 ± 2.08	38.87 ± 2.03	0.660
CAT	1.66 ± 0.13	1.68 ± 0.09	1.63 ± 0.17	1.58 ± 0.11	0.944
GSH-PX	621.60 ± 14.96	627.20 ± 17.51	619.20 ± 10.59	615.20 ± 18.69	0.958
T-SOD	122.12 ± 6.37	122.66 ± 4.50	121.71 ± 5.84	121.43 ± 8.93	0.999
T-AOC	0.32 ± 0.01	0.33 ± 0.03	0.33 ± 0.03	0.31 ± 0.01	0.969
MDA	1.60 ± 0.05	1.57 ± 0.06	1.63 ± 0.06	1.67 ± 0.26	0.962
TG	0.22 ± 0.03	0.23 ± 0.04	0.21 ± 0.02	0.25 ± 0.03	0.783
CHO	1.80 ± 0.11	2.01 ± 0.12	1.67 ± 0.07	1.96 ± 0.13	0.149
HDL-C	1.14 ± 0.07	1.29 ± 0.09	1.06 ± 0.06	1.21 ± 0.10	0.234
LDL-C	0.32 ± 0.03	0.35 ± 0.03	0.32 ± 0.01	0.40 ± 0.03	0.160
IgA	4.57 ± 0.75	4.88 ± 0.44	4.46 ± 0.68	4.51 ± 0.19	0.951
IgM	160.43 ± 1.97	161.70 ± 4.02	155.81 ± 2.55	156.44 ± 2.23	0.383
IgG	189.35 ± 9.85	190.21 ± 7.28	193.06 ± 7.67	184.60 ± 6.89	0.900
BUN	7.87 ± 0.41	8.28 ± 0.46	7.60 ± 0.46	8.69 ± 0.34	0.302

GLU, glucose; TP, total protein; ALB, albumin; GLB, globulin; ALP, alkaline phosphatase; AST, aspartate aminotransferase; ALT, alanine aminotransferase; BUN, blood urea nitrogen; TG, triglyceride; CHO, total cholesterol; HDL-C, high-density lipoprotein cholesterol; LDL-C, low-density lipoprotein cholesterol; CAT, catalase; GSH-PX, glutathione peroxidase; T-SOD, total superoxide dismutase; T-AOC, total antioxidant capacity; MDA, malondialdehyde; IgA, immunoglobulin A; IgM, immunoglobulin M; IgG, immunoglobulin G.

**Table 7:** Effects of fermented rapeseed meal at different replacement levels on α-diversity indexes in sika deer during the pre-antler growth period (mean ± SE, *n* = 6).

Items	Chao1	Shannon	Observed_Species
CK	3648.39 ± 116.75	10.39 ± 0.09	3454.67 ± 99.34
L-FRSM	3814.62 ± 122.17	10.43 ± 0.09	3519.67 ± 108.23
M-FRSM	3520.06 ± 98.85	10.36 ± 0.06	3333.83 ± 85.92
H-FRSM	3576.31 ± 60.40	10.42 ± 0.04	3343.00 ± 51.11
*p*-values	0.231	0.901	0.403

## Data Availability

The raw data supporting the conclusions of this article will be made available by the authors on request.

## Supplementary Materials

The following supporting information can be downloaded at: https://www.mdpi.com/article/10.3390/ani16081221/s1, Figure S1: KEGG functional prediction of unique OTUs in each experimental group based on PICRUSt2 analysis.
