# Supplementary material for: Effects of Fermented Rapeseed Meal as a Substitute for Soybean Meal on Growth Performance, Nutrient Digestibility, Serum Biochemical Indices and Gastrointestinal Microbiota of Sika Deer (Cervus nippon) During the Pre-Antler Growth Period
Source: Animals (Basel). 2026 Apr 16;16(8):1221. doi: 10.3390/ani16081221 (PMC13113292; doi:10.3390/ani16081221)
Supplement: Supplementary file 1 [file animals-16-01221-s001.zip › animals-4186815-supplementary.pdf]

Supplementary materials

Effects of Fermented Rapeseed Meal as a Substitute for Soybean Meal on Growth Performance, Nutrient Digestibility, Serum Biochemical Indices and Gastrointestinal Microbiota of Sika Deer (*Cervus nippon*) During the Pre-Antler Growth Period

Jiaxin Tian [1], Hui Zhao [1], Qiaoru Zhang [1], Haoran Sun [1], Zuer Gao [1], Luyang Sun [1], Chengzhi Zhu [1], Fansheng Kong [1], Xiuhua Gao [2], Qingkui Jiang [3,*] and Tietao Zhang [1,4,*]

1. Institute of Special Animal and Plant Sciences, Chinese Academy of Agriculture Sciences, Changchun 130112, China; tjx0813@126.com (J.T.); baobeihuihui815@163.com (H.Z.); sarahzhang96@163.com (Q.Z.); solomoncat@163.com (H.S.); gze1010@163.com (Z.G.); 17767877397@163.com (L.S.); 13174428551@163.com (C.Z.); 15562382821@163.com (F.K.)
2. Institute of Feed Research, Chinese Academy of Agriculture Sciences, Beijing 100081, China; xiuhuagao@126.com
3. Public Health Research Institute, New Jersey Medical School, Rutgers Biomedical and Health Sciences, Rutgers, The State University of New Jersey, Newark, NJ 07103, USA
4. National Nanfan Research Institute, Chinese Academy of Agricultural Sciences, Sanya 572100, China

* Correspondence: qj35@njms.rutgers.edu (Q.J.); zhangtietao@caas.cn (T.Z.)

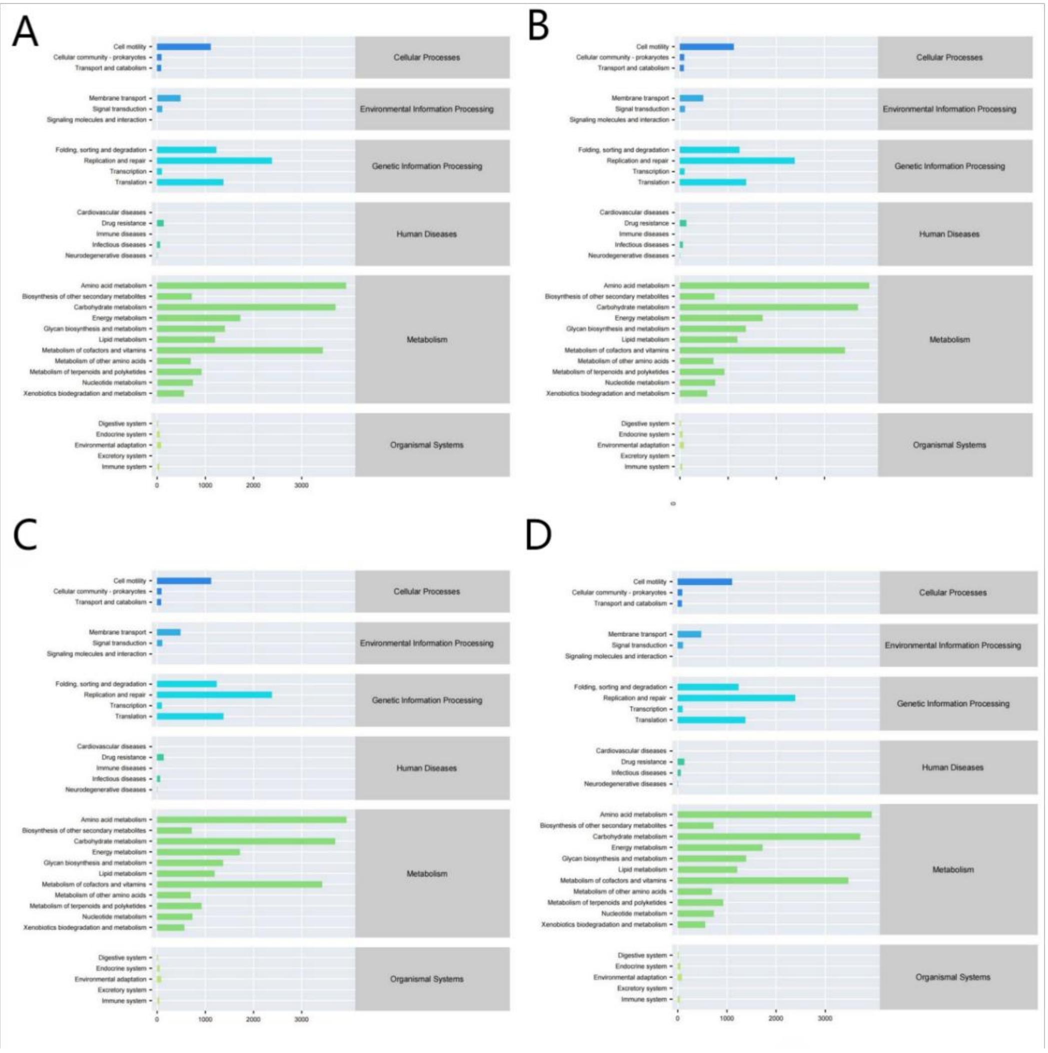

Figure S1 KEGG functional prediction of unique OTUs in each experimental group based on PICRUSt2 analysis.

Functional profiles were predicted using PICRUSt2 from the unique operational taxonomic units (OTUs) identified in each group and annotated against the KEGG database at level 2 pathways. Panels **A–D** represent the functional distribution of unique OTUs in the CON, L-FRSM, H-FRSM, and H-FRSM groups, respectively. The predicted functions are categorized into major KEGG pathway classes, including Cellular Processes, Environmental Information Processing, Genetic Information Processing, Human Diseases, Metabolism, and Organismal Systems. The x-axis represents the relative abundance of predicted functional genes, while the y-axis shows the KEGG level-2 functional categories. Overall, the functional composition of predicted pathways derived from unique OTUs was largely similar among groups, with metabolism-related pathways (e.g., amino acid metabolism, carbohydrate metabolism, and energy metabolism) being the most abundant categories.
